# Determining the Extent and Characterizing Coral Reef Habitats of the Northern Latitudes of the Florida Reef Tract (Martin County)

**DOI:** 10.1371/journal.pone.0080439

**Published:** 2013-11-25

**Authors:** Brian K. Walker, David S. Gilliam

**Affiliations:** 1 Nova Southeastern University, Oceanographic Center, Dania Beach, Florida, United States of America; 2 Nova Southeastern University, Oceanographic Center, Dania Beach, Florida, United States of America; New England Aquarium, United States of America

## Abstract

Climate change has recently been implicated in poleward shifts of many tropical species including corals; thus attention focused on higher-latitude coral communities is warranted to investigate possible range expansions and ecosystem shifts due to global warming. As the northern extension of the Florida Reef Tract (FRT), the third-largest barrier reef ecosystem in the world, southeast Florida (25–27° N latitude) is a prime region to study such effects. Most of the shallow-water FRT benthic habitats have been mapped, however minimal data and limited knowledge exist about the coral reef communities of its northernmost reaches off Martin County. First benthic habitat mapping was conducted using newly acquired high resolution LIDAR bathymetry and aerial photography where possible to map the spatial extent of coral reef habitats. Quantitative data were collected to characterize benthic cover and stony coral demographics and a comprehensive accuracy assessment was performed. The data were then analyzed in a habitat biogeography context to determine if a new coral reef ecosystem region designation was warranted. Of the 374 km^2^ seafloor mapped, 95.2% was Sand, 4.1% was Coral Reef and Colonized Pavement, and 0.7% was Other Delineations. Map accuracy assessment yielded an overall accuracy of 94.9% once adjusted for known map marginal proportions. Cluster analysis of cross-shelf habitat type and widths indicated that the benthic habitats were different than those further south and warranted designation of a new coral reef ecosystem region. Unlike the FRT further south, coral communities were dominated by cold-water tolerant species and LIDAR morphology indicated no evidence of historic reef growth during warmer climates. Present-day hydrographic conditions may be inhibiting poleward expansion of coral communities along Florida. This study provides new information on the benthic community composition of the northern FRT, serving as a baseline for future community shift and range expansion investigations.

## Introduction

Effective marine resource management begins with knowing the types, amounts, and spatial distribution of resources. Rigorously ground-truthed benthic habitat mapping via geographic information systems (GIS), a process by which remote sensing data are interpreted into seafloor habitats, provides this valuable information. Globally, benthic habitat mapping has been employed in many coral reef ecosystems, utilizing various techniques and data types including the interpretation of aerial photography, satellite imagery, bathymetric data, *in situ* visual imaging, or a combination thereof [1]. Currently across the ten United States coral reef jurisdictions, over 12,100 km^2^ of shallow-water (< 30 m) coral reef habitats have been mapped by this process [2].

Coral reefs thrive in warm tropical waters, therefore much of the coral reef habitat mapping has focused on tropical and subtropical areas with little regard for higher latitude temperate regions even though coral communities may be present [3], [4]. Climate change has recently been implicated in poleward shifts of many tropical species including corals [5], [6], [7], thus attention focused on higher latitude coral communities is warranted to investigate possible range expansions and ecosystem shifts due to global warming.

As the northern extension of the Florida Reef Tract (FRT), southeast Florida is a prime region to study climate change effects. The FRT, the third largest barrier reef ecosystem in the world [8], [9], spans approximately 595 km of linear coastline from the Dry Tortugas in the southwest to Martin County in the northeast. The 135 km southern portion resides in an east-west orientation mostly at the same latitude (24.5° N) before it arcs northeast over a 245 km span (25.5° N). The final 215 km extends north to 27.25° N. This northern extension transitions from a tropical to temperate Holdridge Life Zone [10] where several estuarine biogeographic zones have been defined [11]. Recent analyses of this northern extension identified several biogeographic spatial barriers where the number of benthic habitats attenuated northward along the coast and various habitat metrics differed significantly between 5 sub-regions [12].

Most of the shallow-water FRT benthic habitats have been mapped [12], [13], however minimal data and limited knowledge exist about the coral reef communities of its northernmost reaches off Martin County. This study maps and characterizes the seafloor in Martin County to provide benthic resource data. First benthic habitat mapping was conducted using newly-acquired high resolution LIDAR bathymetry and aerial photography where possible to map the spatial extent of coral reef habitats. Quantitative data were collected to characterize benthic cover and stony coral demographics and a comprehensive accuracy assessment was performed. The benthic mapping data were then analyzed in the habitat biogeographic context of Walker [12] to determine if the newly mapped habitat types and configurations differ from those found further south. These data not only provide new information on the little-studied benthic community composition, but they also serve as a baseline for future community shift and range expansion investigations, assist resource managers in the development of conservation action strategies, and enable permitted activity impact avoidance enforcement.

## Methods

No specific permissions were required for this study. The study was observatory and did not include the disturbing or removal of organisms other than those expected from normal SCUBA diving. The study was approved by Florida Department of Environmental Protection, Florida Fish and Wildlife Conservation Commission, and the National Oceanic and Atmospheric Administration, with the latter two providing the funding. A portion of the study included the St. Lucie Inlet Preserve State Park, who also supported the effort. The field studies did not involve endangered or protected species at the time of the study.

### 2.1 Benthic habitat mapping

The marine benthic habitats in Martin County were mapped using a combined technique approach similar to other southeast Florida counties [1], [12]. The area of interest covered approximately 350 km^2^ of seafloor from shore to the 30 m depth contour. Image-based analyses in deeper water were not useful due to poor water clarity; therefore, a high resolution (4 m) LIDAR bathymetric survey was conducted to image the sea floor. LIDAR were acquired in December 2008 and 2009 by Blom Aerofilms, Ltd. using an Airborne Hydrography AB Hawkeye II LIDAR [14]. The data were collected with a hydrographic accuracy of ± 2.5 m horizontal and ± 0.25 m (rms) vertical (IHO order 1) at an altitude of approximately 500 m, yielding a point spacing of approximately 4 m. Cleaned and processed LIDAR point data were then interpolated by nearest neighbor into high resolution digital elevation models and hillshaded surfaces.

Benthic habitat maps were produced by visual interpretation of the bathymetric LIDAR, aerial photography, and other data at a 1∶6000 scale with a 0.4 hectare minimum mapping unit, classifying seafloor features based on their geomorphology. Geomorphology and depth were used as surrogates for differing benthic communities [15] based on previous regional mapping efforts [1], [16], [17] and supplemental information. A comprehensive dataset from previous work at the county, state, and federal level was assembled in ArcGIS to aid in the seafloor feature identification. The high resolution hillshaded LIDAR images were the primary data source used to discriminate seafloor features. Additionally the interpretation was supplemented by other datasets including Martin County Property Appraisal aerial photography, Southeast Florida Coral Reef Evaluation and Monitoring Program monitoring data, and FWRI artificial reef location data. Conflicts between data types were resolved by expert-driven interpretation based on the agreement of the majority of data types with an emphasis on the most recent data.

### 2.2 Classification scheme

The benthic habitat classifications conformed to the scheme used in previous regional efforts [1], [12] which were adopted from NOAA hierarchical classification scheme used in Puerto Rico and the U.S. Virgin Islands NOAA Technical Memorandum National Ocean Service (NOS) National Centers for Coastal Ocean Science (NCCOS) Center for Coastal Monitoring & Assessment CCMA 152 [18], [19] with some modification. The habitats identified in the mapping were as follows:

**Coral reef and colonized hardbottom.** Hardened substrate of unspecified relief formed by the deposition of calcium carbonate by reef building corals and other organisms or existing as exposed bedrock. Habitats within this category have some colonization by live coral.

**Colonized pavement.** Flat, low-relief, solid carbonate rock with coverage of macroalgae, hard coral, gorgonians, and other sessile invertebrates that are dense enough to partially obscure the underlying carbonate rock.

**Colonized pavement-shallow.** Colonized pavement in water shallower than 10 m. This category includes rubble in many areas; however, consolidated rubble fields are a less frequent feature in shallow water. Especially inshore of the ridge complexes, limited rubble is found and a wide, contiguous area of pavement is encountered. This area can have variable sand cover, which shifts according to wave energy in response to weather. Thus, some of the colonized pavement will always be covered by shifting sand and the density of colonization will be highly variable.

**Ridge.** Linear, shore-parallel, low-relief features that appear to be submerged cemented ancient shoreline deposits. Presumably, they are an extension of the foundation upon which the linear reefs grew further south and consist of early Holocene shoreline deposits; however, verification is needed. The biological cover is similar to that of colonized pavement with macroalgae, scleractinians, gorgonians, and other sessile invertebrates that are dense enough to partially obscure the underlying carbonate rock.

**Ridge-deep.** Linear, often shore-parallel, low-relief features that mostly occur deeper than 20 m. It consists of hardbottom with sparse benthic communities in most parts likely due to variable and shifting rubble and sand cover. Some parts contain exposed ledges where large fish (e.g. Goliath grouper, Nurse Shark) may congregate.

**Ridge-shallow.** Ridges found in water shallower than 10 m near shore that are geomorphologically distinct, yet their benthic cover remains similar to the shallow colonized pavement communities on the surrounding hard grounds.

**Deep ridge complex.** A complex of ridges found in deep water in northern Palm Beach and Southern Martin Counties. These features reside in depths from 20 to 35 m and are presumed to be of cemented beach dune origin. Most of this habitat consists of low cover, deep communities dominated by small gorgonians, sponges, and macroalgae, but denser areas exist, especially near areas of higher relief. Some areas, particularly between ridges, may contain large areas of shifting unconsolidated sediments.

**Scattered rock in unconsolidated sediment (SCRUS)-deep.** Primarily sand bottom with scattered rocks that are too small to be delineated individually in water deeper than 20 m.

**SCRUS-shallow.** Primarily sand bottom with scattered rocks that are too small to be delineated individually in water shallower than 20 m.

**Unconsolidated sediments.** Unconsolidated sediment with less than 10 percent cover of submerged vegetation.

**Sand.** Coarse sediment typically found in areas exposed to currents or wave energy.

**Sand–deep.** Sand deeper than the 25 m contour exposed to a lower energy environment that can have finer grain size, sparse *Halophila* spp., and a rubble component. This habitat can contain a high cover of turf and low-lying benthos in some areas.

**Sand–shallow.** Shallow water (< 25 m) sediment exposed to a higher energy environment. Large, mobile sand pockets are found on the areas of consolidated hardgrounds. It is believed that the sand movement is a deciding factor in the generation of benthic patterns.

### Other delineations

**Artificial.** Manmade submerged habitats such as wrecks, portions of rip-rap jetties, and spoil piles.

**Inlet jetty.** Artificial structures placed at the inlet channel primarily to block wave energy and reduce erosion.

**Sand borrow area.** Pits excavated during previous sand dredging projects for beach nourishment.

### 2.3 *In situ* benthic characterization

Benthic characterization surveys were conducted in August 2012. Site locations were determined by a statistically robust random sample design similar to Smith *et al*. [20] stratifying across habitat classes throughout the county. The sites were distributed across the seascape to provide data on all the main hardbottom habitats and account for latitudinal variation. The data collection methods were adopted from those used in the Mesoamerican Barrier Reef System Project [21] and the widely used Atlantic and Gulf Rapid Reef Assessment [22]. Data at each site were collected on four 30 meter point-intercept transects at an intercept density of 0.25 m for a total of 480 (120×4) points per site. At each point, divers identified the organism under the transect tape by major functional groups (hard coral species, turf algae, macroalgae, sponge, zoanthid, etc.) or bare substrate type. As underwater dive limits permitted, all stony corals within 0.5 m of either side of the transects were recorded for colony size (length, width, height), live tissue area (length x width of live tissue), percent mortality, presence of bleaching, and presence of disease. Finally rugosity was estimated along each transect by measuring the distance along the bottom contour to the linear distance. All four measurements were combined to create a rugosity index for each site by dividing the contour distance by the linear distance.

Multivariate analyses were performed in Primer v6. A cluster analysis and corresponding non-metric multi-dimensional scaling (MDS) plot was constructed using Bray-Curtis similarity indices of the benthic cover data (square-root transformed) to evaluate benthic cover between sites. A one-way analysis of similarity (ANOSIM) was performed to statistically determine the strength of the site categorization by habitat. ANOSIM is a permutation-based hypothesis test analogous to univariate analyses of variance (ANOVAs) that tests for differences between groups of (multivariate) samples from different experimental treatments. The closer the R statistic is to 1, the stronger the categorical groups. Its strength is dependent on the number of samples per category which defines the number of possible permutations. One-way nonparametric ANOVA using the Wilcoxon method was used to examine differences in rugosity and biological cover category data (i.e., the number of major live functional group categories per site) by habitat.

### 2.4 Map accuracy assessment

A map accuracy assessment (AA) was performed. Target locations were determined by a GIS-based, stratified random sampling technique used in other regional mapping efforts [17], [23], [24]. The map proportions of all Coral Reef and Colonized Hardbottom and Artificial habitats were used to determine the percentage of assessment sites per habitat. An additional 33 locations were added to sand which is comparable to other efforts. Four benthic habitat classes found in the draft benthic habitat map were excluded from the accuracy analysis; the Inlet Jetty, Sand Borrow Areas, Sand-Deep, and Deep Ridge Complex. The first two were excluded because they are unnatural habitats, although artificial was included because of their ecologic value. The Deep Ridge Complex was excluded because it was mapped and assessed during the Palm Beach mapping effort [16]. This yielded 199 stratified random accuracy assessment target locations to be visited by drop camera and analyzed by confusion matrix approach [25].

Underwater video from a drop camera was taken at each AA target location. This procedure involved the boat positioning itself within 5 m of the target and lowering a Sea Viewer 950 underwater color video drop camera with a Sea-trak GPS video overlay connected to a Garmin 76CSx GPS with WAAS correction (<3 m accuracy) to the bottom. Color video was recorded over the side of the stationary/drifting vessel approximately 0.5–2 m from the seafloor. Fifteen second to two minute video clips were recorded directly to a digital video recorder. Video length depended on the habitat type and vessel drift. Videos of large expansive sand habitats were generally short (< 1 min) while reef habitats, especially edges, were longer. Concurrent with recording video, an observer categorized each site according to the video and surrounding area into a database.

Statistical analyses to determine the thematic accuracy by confusion matrix approach were derived from Congalton [26], Hudson and Ramm [27], and Ma and Redmond [28]. Matrices of user and producer map accuracy error, overall map accuracy error, and the Tau coefficient were generated. The error matrices were constructed as a square array of numbers arranged in rows (map classification) and columns (true, or ground-truthed classification). The overall accuracy (P_o_) was calculated as the sum of the major diagonal (i.e. correct classifications) divided by the total number of accuracy assessment samples. The producer’s and user’s accuracies are both category-specific. Each diagonal element was divided by the column total to yield a producer’s accuracy and by the row total to yield a user’s accuracy. The producer’s and user’s accuracies provide different perspectives on the classification accuracy of a map. The producer’s accuracy (omission/exclusion error) indicates how well the mapper classified a particular habitat (e.g. the percentage of times that substrate known to be sand was correctly mapped as sand). The user’s accuracy (commission/inclusion error) indicates how often map polygons of a certain habitat type were classified correctly (e.g. the percentage of times that a polygon classified as sand was actually sand). The Tau coefficient (T_e_) is a measure of the improvement of classification accuracy over a random assignment of map units to map categories [28]. In this case, T_e_ is simply an adjustment of P_o_ by the number of map categories. As the number of categories increases, the probability of random agreement diminishes, and T_e_ approaches P_o_.

Direct interpretation of producer’s and overall accuracies can be problematic, as the stratified random sampling protocol can potentially introduce bias [29], [30], [31]. Stratification ensures adequate representation of all map categories, by assigning an equal number of accuracy assessment to each map category. This caused small extent map categories to be sampled at a greater density (observations per unit area) than large ones. The bias introduced by differential sampling rates was removed using the method of Card [32], which utilizes the known map marginal proportions, i.e. the relative areas of map categories. The map marginal proportions were calculated as the area of each map category divided by the total area calculated from the Martin County habitat map polygons. The map marginal proportions were also utilized in the computation of confidence intervals for the overall, producer’s, and user’s accuracies [32].

### 2.5 Spatial analyses

Benthic habitat polygons were tested for spatial autocorrelation in ArcGIS using Moran’s Index to ensure the polygons did not significantly differ from a random distribution. Map data were then combined with the previous southeast Florida maps [12] and statistically examined to determine where the number and size of seagrass, coral reef, and colonized hardbottom habitats significantly differ. Two hundred and forty-eight parallel, cross-shelf vector-line transects spaced 750 m apart were created in GIS throughout the entire mapped region. An intersect was performed between the vector-line transects and the benthic habitat polygons, which broke the transect lines at each point where they intersected with a habitat polygon. The length of each resulting line segment was calculated to determine the linear cross-shelf distance of each habitat (width). A cluster analysis and corresponding non-metric multi-dimensional scaling (MDS) plot was then constructed using Bray-Curtis similarity indices (PRIMER v6) of the cross-shelf habitat width data (square-root transformed) to evaluate regions with distinct habitat composition. The groups of transects that occurred within the clusters with 60% similarity were then categorized in GIS and visually examined to evaluate the clusters for any spatial grouping consistency. Inspection of the benthic habitats where MDS clusters split helped identify the key locations in the habitat mapping data where the regional boundaries were defined. After defining the boundaries, all cross-shelf transects were categorized by the corresponding region. These categories were imported in Primer as factors and a one-way analysis of similarity (ANOSIM) was performed to statistically determine their similarity. The factors were also displayed on the MDS plot to see how the categorization related to the 60% MDS clusters.

## Results

### 3.1 Habitat extents

**Figure 1:**
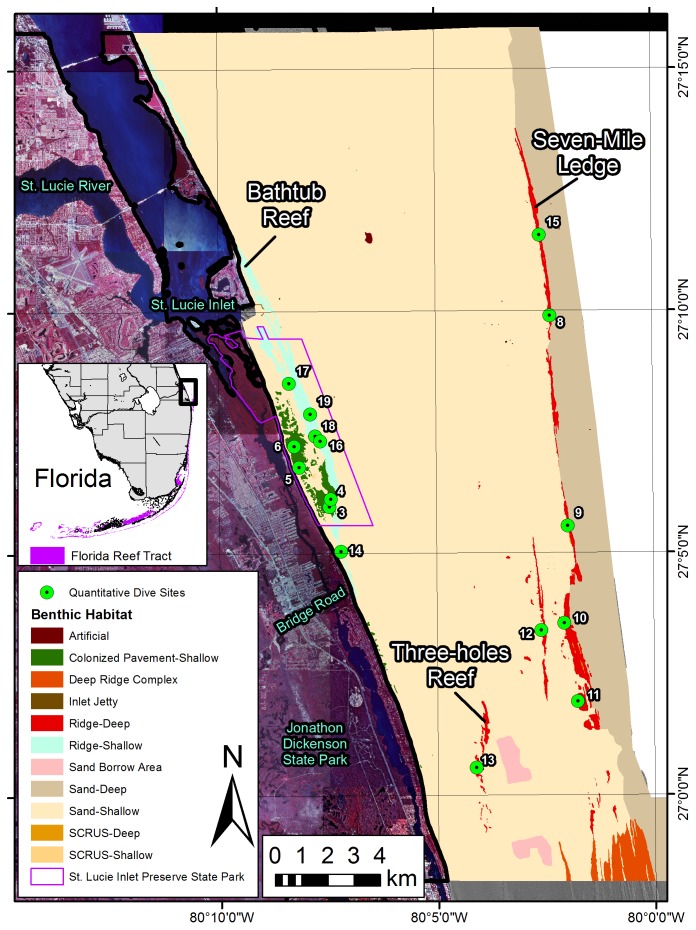
Martin County quantitative ground validation sites overlaying the benthic habitat polygons. Dives at sites 1, 2, and 7 were abandoned due to strong current. Data sources: Land imagery is 2000 USGS Digital Orthophoto Quads mosaicked and provided by the South Florida Water Management District. The habitat map was a result of this study. Grey hill-shaded lidar data were collected by Tenix LADS in 2002 and provided by Palm Beach County Environmental Resource Management.

Planar area of the mapping effort totaled 374.4 km^2^ in GIS of which 95.2% was Sand, 4.1% was Coral Reef and Colonized Pavement, and 0.7% was Other Delineations (Table 1). Hardbottom habitats were sparse outside of a shallow, near shore area around St. Lucie Inlet and a few thin deep ridge lines (Figure 1). Although not confirmed by coring, these features are thought to be cemented beach dunes submerged during the last Holocene sea level transgression [12], [33]. The most extensive deep hardbottom was the northern end of the Deep Ridge Complex which extends from Palm Beach into southern Martin for about 2 km before it is covered with sediment. Only small, thin portions of the tallest ridges are exposed further north. In southern Martin there are three shore-parallel deep ridge lines. The first deep ridge, known as Three-Holes reef, is located approximately 2 km from shore in 18 m water depth and extends approximately 3.5 km northward in a mostly continuous arrangement. The second deep ridge appears at the same latitude that Three-Holes terminates, but it is approximately 6 km from shore in 22 m of water. This mostly continuous feature extends northward for about 6 km. The third deep ridge, known as Seven-Mile-Ledge, is the most conspicuous deep (22 m) hardbottom feature. Despite its name, this feature is located approximately 6 km (∼ 4 miles) from shore in southern Martin. This is also its widest portion at just about 0.5 km. This ridge extends northward over 23 km with relatively few (4) small breaks or gaps. At its northern terminus, it is located about 12.8 km (8 miles) from shore in 25 m water depth.

**Table 1:** Martin County Benthic Habitat Areas (km^2^).

Habitat	Type	Modifier	Modifier Area (km^2^)			Type Area (km^2^)			Habitat Area (km^2^)		
Coral Reef and Colonized Hardbottom	Colonized Pavement	Shallow	2.41	;	0.64%	2.41	;	0.64%	15.45	;	4.13%
	Ridge	Deep	5.11	;	1.36%	12.96	;	3.46%			
		Shallow	4.57	;	1.22%						
		Deep Ridge Complex	3.28	;	0.88%						
	Scattered Coral/Rock in Sand	Deep	0.05	;	0.01%	0.05	;	0.01%			
		Shallow	0.03	;	0.01%	0.03	;	0.01%			
Unconsolidated Sediment	Sand	Deep	42.55	;	11.36%	356.49	;	95.21%	356.49	;	95.21%
		Shallow	313.95	;	83.85%						
Other Delineations	Artificial		0.12	;	0.03%	0.12	;	0.03%	2.49	;	0.66%
	Inlet Jetty		0.02	;	0.00%	0.02	;	0.00%			
	Sand Borrow Area		2.35	;	0.63%	2.35	;	0.63%			
Total Mapped Area (km^2^)									374.43	;	100.00%

The majority of shallow hardbottom habitats exists near St. Lucie inlet (Figure 1). This is comprised of two habitats, Colonized Pavement-Shallow and Ridge-Shallow. The differences between their delineations were mainly morphological. The Ridge-Shallow has an obvious linear morphology with higher relief at the feature scale (1–10 ha). The Colonized Pavement-Shallow is typically lower relief and has no distinct linear morphology. The combination of these two habitats is referred to as the Nearshore Ridge Complex [12], [33]. The shallow Martin County ridges extend 2.5 km north of the inlet and 11.5 km south in a shore-parallel orientation. The eastern side resides in about 10 m depth, it crests near 3 m and the western side remains shallow in some parts and drops back to 10 m in others. The Colonized Pavement-Shallow is located westward of the shallow ridge in waters 10 m to 4 m deep, sloping upward toward shore. These habitats terminate at the shoreline. The northern terminus is known as Bath Tub Reef and the southern end is covered by the shoreline just off Bridge Road on Jupiter Island. Small portions of shallow ridge appear north of the inlet off Jensen Beach.

Approximately 356.5 km^2^ were identified as unconsolidated sediments part of which contained different sediment features that were not part of the mapping. The most conspicuous features were large sand dunes throughout the county extending to the northeast. In the south, these dunes appear to be partially or totally burying portions of deep ridge habitats and can be 11 m high extending over 3.6 km wide [34]. Little is known about the movement of these features, but given the dynamic environment and the frequency of high currents, they may be migrating across the seafloor, including over the deep ridges.

### 3.2 Benthic communities

**Figure 2:**
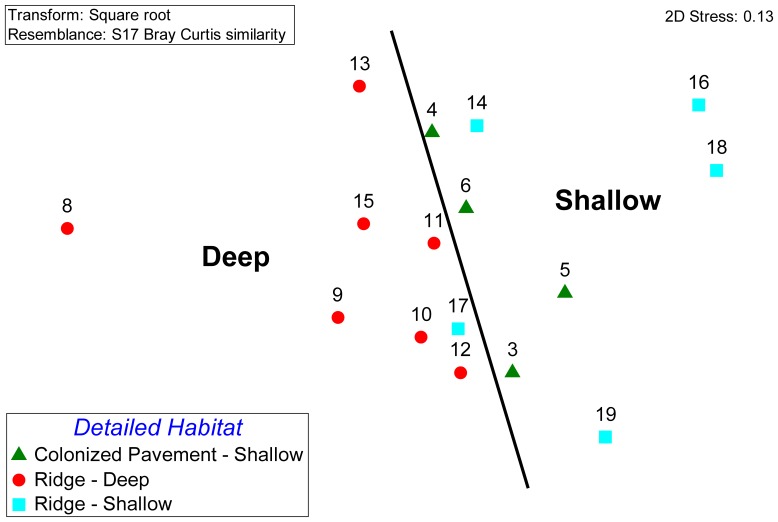
Multidimensional scaling plot of percent cover data for all benthic characterization surveys.

Quantitative benthic characterization data were collected on 16 sites: 7 Ridge-Deep sites, 5 Ridge-Shallow sites, and 4 Colonized Pavement-Shallow sites (Figure 1). A cluster analysis and corresponding non-metric multi-dimensional scaling (MDS) plot showed that the sites were more similar than not, yet subtle distinctions were evident when the sites were categorized by habitat (Figure 2). The Ridge-Deep sites all plotted on one side of the graph and the two shallow habitats on the other, showing there are likely differences between shallow and deep habitats. Furthermore apart from one site, colonized pavement and shallow ridge did not cluster, indicating a wide range of benthic communities between shallow sites.

Multivariate differences of cover types and amounts among sites were not statistically strong among the habitat categories. A one-way analysis of similarity (ANOSIM) indicated the Ridge-Deep and Ridge-Shallow were significantly different (p  =  0.03), supporting the MDS results, yet the difference was not very strong (R  =  0.257) (Table 2). Comparisons between Deep-Ridge and Colonized Pavement-Shallow and between Colonized Pavement-Shallow and Ridge-Shallow were not significant.

**Table 2:** One way analysis of similarity (ANOSIM) results of benthic cover data by habitat types.

ANOSIM Pairwise Tests	R	Significance
Benthic Habitat Groups	Statistic	Level %
**Ridge - Deep, Ridge - Shallow**	**0.257**	**3.2**
Colonized Pavement - Shallow, Ridge - Deep	0.159	19.4
Colonized Pavement - Shallow, Ridge - Shallow	0.038	38.9

Bold type indicates a significant difference between groups. The R statistic indicates the strength of the difference where 1 is the strongest and 0 is weakest.

**Figure 3:**
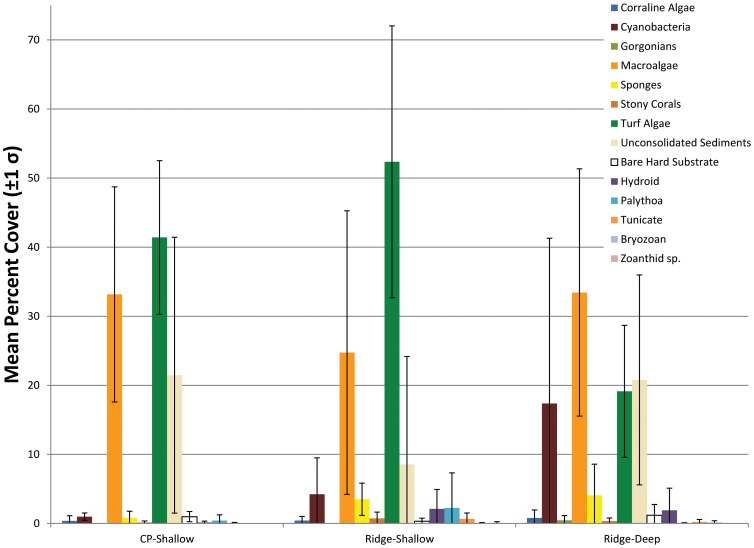
Percent benthic cover data averaged across all sites in the same mapped habitat. Error bars represent one standard deviation.

Differences of mean percent benthic cover by habitat were evident, however, cover varied greatly within habitats and most cover types were low (< 5%) (Figure 3). Turf algae were more abundant on the shallow colonized pavement (41.4%±11.1) and ridge (52.4%±19.6) than the deep ridge (19.1%±9.5) and vice versa for cyanobacteria. Sediment on Colonized Pavement - Shallow sites ranged from < 5% to over 30% and macroalgal cover varied from 17.9% to 53.8%. On the Ridge-Shallow sites, macroalgae varied between 6.3% and 49.4%; Sediment ranged from 0% to 36.3%; cyanobacteria ranged from 0.8% to 13.3%; and the zooanthid Palythoa caribaeorum was only found at one site but contributed 11.3%. The same was true in the Ridge-Deep where macroalgae ranged from 11.9% to 56.7%, sediment ranged from 6% to 49.8%, and cyanobacteria ranged from 3.3% to 69.6%. Cyanobacteria cover on the Ridge-Deep was significantly higher (17.37±6.2 SEM) than Colonized Pavement-Shallow (0.98±8.3 SEM) (p  =  0.01).

Although the mean biotic cover categories (e.g. macroalgae, hydroids, coral) was smaller on the Colonized Pavement-Shallow (5.5±0.84 SEM), it was not significantly different from the Ridge-Shallow (7±0.75 SEM) and Ridge-Deep (7.4±0.64 SEM). However, mean rugosity of the Ridge-Shallow (1.25±0.07 SEM) and Colonized Pavement-Shallow (1.09±0.08 SEM) sites were significantly higher than the Ridge-Deep (1.04±0.06 SEM) (p  =  0.03) indicating the Ridge-Deep sites were flatter.

A total of 553 stony coral colonies were identified, counted, and measured. Nine species were found (Pseudodiploria clivosa, Isophyllia sinuosa, Madracis decactis, Millepora alcicornis, Porites astreoides, Oculina diffusa, Siderastrea siderea, Solenastrea hyades, and Stephanocoenia intersepta), but Siderastrea siderea (80.3%) and O. diffusa (15.9%) dominated the populations. Stony coral density for all sites out of 1737 m^2^ surveyed was 0.32 m^−^^2^, equating to one coral every 3.1 m^2^. Although many corals were counted, colony size was generally small. The estimated total area of live tissue (max length * max width) – (max length * max width * percent total mortality) for all 553 colonies was 2.8 m^2^. Three species accounted for 97.7% of the total live coral tissue in the transects; P. clivosa (42.9%), Siderastrea siderea (30.2%), and O. diffusa (24.6%). Although only 8 P. clivosa colonies were counted, they were the largest colonies and accounted for the most live tissue area. Interestingly, S. siderea had the smallest mean length (4.7 cm), yet was the second highest contributor to live tissue area because of its high numbers (444).

Coral density and live tissue area varied between species by habitat. Although not significant due to high variation, Ridge – Deep habitats had the highest mean coral density (x^−^ = 0.48±0.11 SEM) followed by Ridge – Shallow (x^−^ = 0.29±0.13 SEM) and Colonized Pavement – Shallow (x^−^ = 0.22±0.14 SEM). *S. siderea* and *O. diffusa* were the densest corals in all habitats. Although not significant, mean *S. siderea* densities were highest in the deep ridge (x^−^ = 0.43±0.10 SEM), then shallow ridge (x^−^ = 0.23±0.12 SEM), and were lowest on the shallow colonized pavement (x^−^ = 0.13±0.14 SEM). Mean *O. diffusa* densities were highest on the shallow colonized pavement (x^−^ = 0.08±0.04 SEM), lower on the deep ridge (x^−^ = 0.05±0.03 SEM), and lowest on the shallow ridge (x^−^ = 0.03±0.04 SEM). Mean estimated live tissue area was not significantly different between habitats. *Pseudodiploria clivosa* had the highest estimated mean coral live tissue, but it was only found in the shallow ridge habitat.

Mean maximum coral length and height were low for most species (less than 10 cm) and did not significantly differ between habitats. There were one 12 cm *P. astreoides* and one 13 cm *S. intersepta*. *P. clivosa* was the largest species found, with a mean max length of 39.1 (±23.2) cm out of 8 colonies that ranged from 33 to 80 cm. Two of these colonies were also the tallest corals encountered (25 cm). *P. clivosa* (11.6±9.9) was the only species whose mean max height was above 10 cm.

### 3.3 Accuracy assessment

The assessment of major habitats yielded a high level of accuracy as indicated by the overall accuracy (85.6%) (Table 3), the overall accuracy adjusted for known map marginal proportions (adjusted accuracy) (94.9%) (Table 4), and the Tau coefficient (0.713). Of the 26 classification errors (which excluded artificial sites), 24 were due to Unconsolidated Sediment being found in polygons classified as Coral Reef/Colonized Hardbottom. This yielded a low producer’s accuracy (63.1%) for soft bottom; however correction to map marginal proportions yielded a much higher result (99.4%). The converse was also true where a high producer’s accuracy for hardbottom (98.3%) was drastically reduced by map proportions (37.8%) due to its low spatial coverage even though only 2 errors were found. The detailed habitat accuracy was slightly lower than major habitat, as indicated by the overall accuracy (85.0%) (Table 5), the overall adjusted accuracy (91.5%) (Table 6), and the Tau coefficient (0.828).

**Table 3:** Error matrix for Major Habitat.

		TRUE ( j )			
	*MAJOR HABITAT*	Hard	Soft	*n_i -_*	USERS Accuracy (%)
**( i ) **	**Hard**	**114**	24	138	**82.6**
**MAP**	**Soft**	2	**41**	43	**95.3**
	***n_- j_***	116	65	**181**	**< = ** ***n***
	**PRODUCERS Accuracy (%)**	**98.3**	**63.1**	**P_o_**	**85.6%**
			**T_e_ = 0.713±0.102**		

The overall accuracy (P_o_) was 85.6%. The Tau coefficient for equal probability of group membership (T_e_) was 0.713, with a 95% Confidence Interval of 0.611– 0.815.

**Table 4:** Error matrix for Major Habitat using individual cell probabilities (P_ij_).

		TRUE ( j )				
	*MAJOR HABITAT*	Hard	Soft	π *_i_*	USERS Accuracy (%)	USERS CI (± %)
**( i ) **	**Hard**	**0.027**	0.006	0.033	**82.6**	**6.5**
**MAP**	**Soft**	0.045	**0.922**	0.967	**95.3**	**6.4**
	***n_- j_***	0.072	0.928	**1.000**	***< = n***	
	**PRODUCERS Accuracy (%)**	**37.8**	**99.4**	**P_o_**	**94.9%**	
	**PRODUCERS CI (± %)**	**32.5**	**0.2**	**CI (±)**	**6.2%**	

The overall accuracy, corrected for bias using the known map marginal proportions (π_i_), was 94.9% with a 95% Confidence Interval of 88.7% – 100%.

**Table 5:** Error matrix for Detailed Habitat.

					TRUE	( j )					
*DETAILED HABITAT*		Colonized Pavement-Shallow	Ridge-Deep	Ridge-Shallow	Scattered Rock in Unconsolidated Sediment-Deep	Scattered Rock in Unconsolidated Sediment-Shallow	Sand-Deep	Sand-Shallow	Artificial	*n_i -_*	USERS Accuracy (%)
	**CP - Shallow**	**26**						1		27	**96.3**
	**Ridge-Deep**		**40**				12	5		57	**70.2**
	**Ridge-Shallow**			**46**				6		52	**88.5**
**( i ) **	**SCRUS - Deep**				**1**					1	**100.0**
**MAP **	**SCRUS - Shallow**					**1**				1	**100.0**
	**Sand-Deep**						**2**			2	**100.0**
	**Sand-Shallow**		2					**39**	2	43	**90.7**
	**Artificial**							1	**9**	10	**90.0**
	***n_- j_***	26	42	46	1	1	14	52	11	**193**	**< = ** ***n***
	**PRODUCERS Accuracy (%)**	**100.0**	**95.2**	**100.0**	**100.0**	**100.0**	**14.3**	**75.0**	**81.8**	**P_o_**	**85.0%**
										**T_e_ = 0.828**	**± 0.058**

The overall accuracy (P_o_) was 85.0%. The Tau coefficient for equal probability of group membership (T_e_) was 0.828, with a 95% Confidence Interval of 0.770 – 0.886. Blank cells indicate 0 occurrences.

**Table 6:** Error matrix for Detailed Habitat using individual cell probabilities (P_ij_).

		TRUE ( j )										
*DETAILED HABITAT*		Colonized Pavement-Shallow	Ridge-Deep	Ridge-Shallow	Scattered Rock in Unconsolidated Sediment-Deep	Scattered Rock in Unconsolidated Sediment-Shallow	Sand-Deep	Sand-Shallow	Artificial	π _i_	USERS Accuracy (%)	USERS CI (± %)
	**CP - Shallow**	**0.00630**						0.00024		0.007	**96.3**	**7.3**
	**Ridge-Deep**		**0.00972**				0.00292	0.00121		0.014	**70.2**	**12.1**
	**Ridge-Shallow**			**0.01097**				0.00143		0.012	**88.5**	**8.9**
**( i ) **	**SCRUS - Deep**				**0.00014**					0.000	**100.0**	**0.0**
**MAP **	**SCRUS - Shallow**					**0.00008**				0.000	**100.0**	**0.0**
	**Sand-Deep**						**0.11537**			0.115	**100.0**	**0.0**
	**Sand-Shallow**		0.03959					**0.77210**	0.03959	0.851	**90.7**	**8.9**
	**Artificial**							0.00003	**0.00030**	0.000	**90.0**	**19.0**
	***n_- j_***	0.006	0.049	0.011	0.000	0.000	0.118	0.775	0.040	**1.000**	**< = ** ***n***	
	**PRODUCERS Accuracy (%)**	**100.0**	**19.7**	**100.0**	**100.0**	**100.0**	**97.5**	**99.6**	**0.7**	**P_o_**	**91.5%**	
	**PRODUCERS CI (± %)**	**7.5**	**22.0**	**10.0**	**0.0**	**0.0**	**1.2**	**9.7**	**1.0**	**CI (±)**	**7.5%**	

The overall accuracy, corrected for bias using the known map marginal proportions (π_i_), was 91.5% with a 95% Confidence Interval of 84.0% – 99.0%. Blank cells indicate 0 occurrences.

### 3.4 Spatial analysis

**Figure 4:**
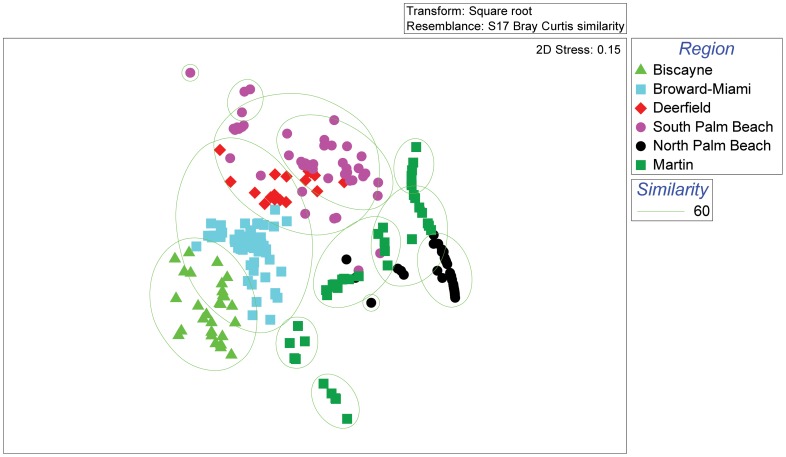
Multidimensional scaling (MDS) plot of Bray-Curtis similarity matrix of 248 regional cross-shelf transects displayed using the six final regional categories. The outlines represent 60% similarity from the cluster analysis.

**Figure 5:**
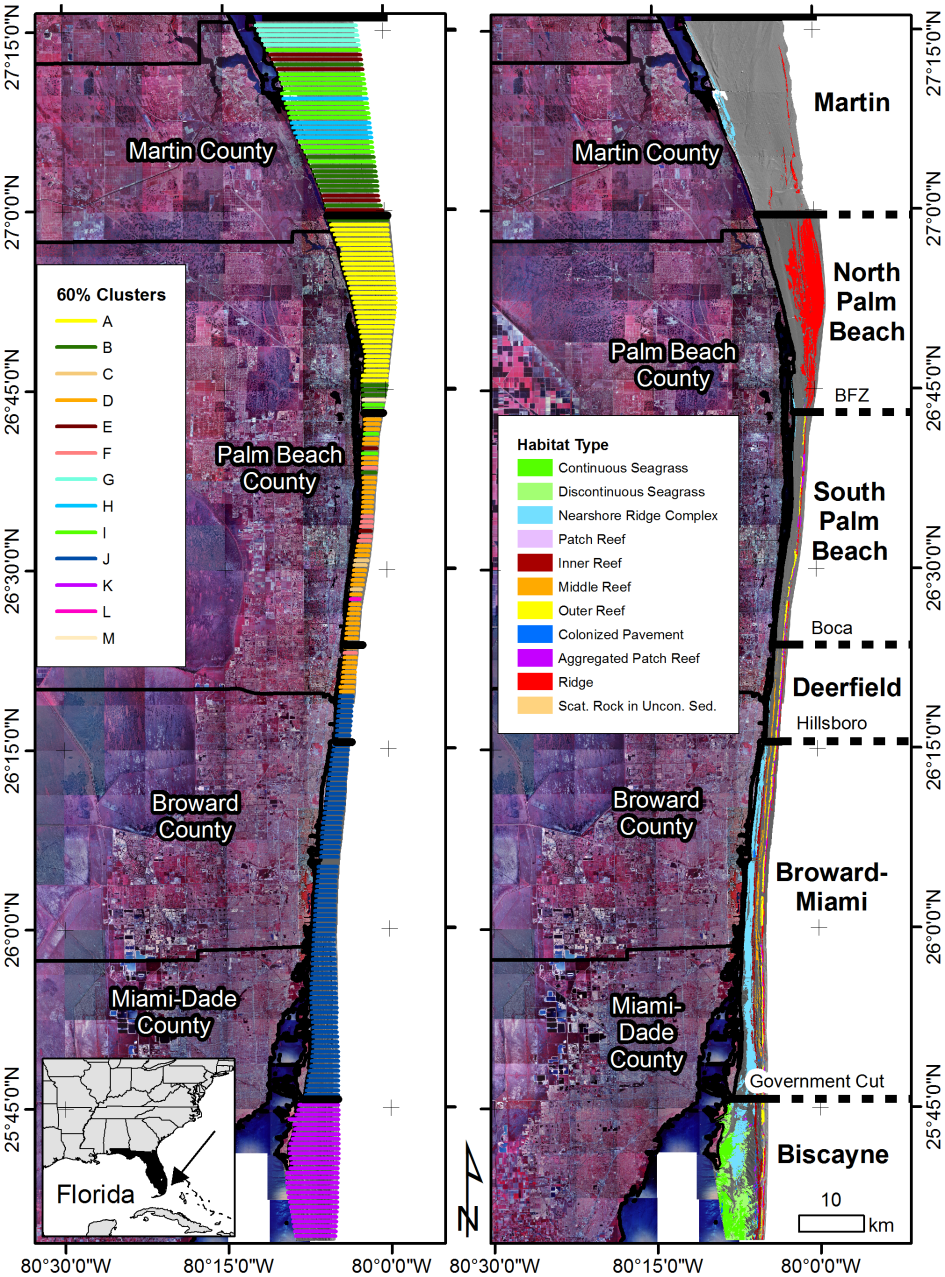
Overview maps showing the cross-shelf transects symbolized by the 60% similarity MDS clusters (left) and the six identified regions (right). BFZ  =  Bahamas Fault Zone. Data sources: Land imagery is 2000 USGS Digital Orthophoto Quads mosaicked and provided by the South Florida Water Management District. The habitat map was a result of this and previous studies by the author (See Walker et al. (2008) and Walker (2012)). Grey hill-shaded lidar data were collected by Tenix LADS in 2001 and 2002. Lidar data were provided by Miami-Dade County Environmental Resource Management, Broward County Natural Resources Planning and Management Division, Palm Beach Environmental Resource Management, and Coastal Planning and Engineering. Martin lidar were collected as part of this study.

Five regions along the coast were previously identified using the same methodology [12]. The purpose of repeating it here was to evaluate if a new transition between North Palm Beach and Martin was warranted. Spatial autocorrelation tests on the benthic habitat polygon areas using Moran's Index did not show a pattern significantly different from random (Moran's I 0.002; z-score 0.08; p-value 0.94). Cluster analysis of the cross-shelf transects yielded 13 clusters at the 60% similarity level and the two dimensional MDS plot showed a medium stress (0.15) (Figure 4). The Biscayne, Broward-Miami, and South Palm Beach region MDS clusters showed spatial groupings similar to the previous study. The Deerfield region, which was the weakest result in the previous study [12], was not evident in this analysis. The North Palm Beach transects clustered into one group that was also spatially clustered (Cluster A in Figure 5). The transects in Martin were members of five MDS clusters, however all but Cluster B were exclusive to the Martin area. This indicates that the seafloor habitat morphology in Martin is distinctly different from areas further south and represents a unique region.

The analysis of similarity (ANOSIM) performed to statistically determine the similarity of the six final regions based on the cross-shelf transect data showed strong differences (R statistic > 0.849) between categories in 11 of the 15 pairwise tests (Table 7). The weakest pairwise comparison was between Deerfield and South Palm Beach regions (R  =  0.115). Although not the strongest, North Palm Beach and Martin comparisons were significantly strong (R  =  0.621) and justified the split. Visual inspection of the transects in GIS revealed that weaknesses in the clusters were likely due to the absence of certain habitats in specific transects that were present at the larger scale, but were not captured along the transect.

**Table 7:** A summary of the analysis of similarity (ANOSIM) pairwise test between the six identified biogeographic regions.

ANOSIM Pairwise Tests	R	Significance
Groups	Statistic	Level %
Biscayne, Broward-Miami	0.941	0.1
Biscayne, Deerfield	0.993	0.1
Biscayne, South Palm Beach	0.873	0.1
Biscayne, North Palm Beach	1	0.1
Biscayne, Martin	0.806	0.1
Broward-Miami, Deerfield	0.895	0.1
Broward-Miami, South Palm Beach	0.883	0.1
Broward-Miami, North Palm Beach	0.998	0.1
Broward-Miami, Martin	0.88	0.1
Deerfield, South Palm Beach	0.115	3.2
Deerfield, North Palm Beach	0.996	0.1
Deerfield, Martin	0.671	0.1
South Palm Beach, North Palm Beach	0.849	0.1
South Palm Beach, Martin	0.531	0.1
North Palm Beach, Martin	0.621	0.1

All tests were significant (p ≤ 0.032). The R statistic indicates the strength of the difference where 1 is the strongest and 0 is weakest.

## Discussion

### 4.1 Coral reef ecosystem regions

Recent analyses of habitat spatial distributions along the southeast Florida coast identified 5 coral reef ecosystem regions and potential biogeographic boundaries [12]. The northern extent of these analyses and maps was in southern Martin County just north of the Deep Ridge Complex. The addition of the Martin County maps to these analyses justified the creation of a sixth region north of the North Palm Beach region based on habitat types and configurations (Figure 5). In contrast to reef regions further south where coral reef habitat areas ranged from 13.93% (South Palm Beach) to 52.6% (Broward-Miami) [12], the Martin area contained 4.1% coral reef habitat, most of which was in a few thin deep and shallow ridges. The types and extent of shallow-water (< 30 m) coral reef habitats in the northern Florida Reef Tract are now known and can be included in the spatial assessment for coral reef ecosystem regions.

Differences in benthic cover indicate that the Martin region has a biological composition different from other areas of the FRT. In 2007, a two-year detailed regional study on macroalgal communities showed that Martin County had the highest macroalgal cover in southeast Florida [35]. Cross-shelf and latitudinal differences were evident in algal populations that were not seen solely by summing up the data for each county. In Martin, the three shallow ridge sites had a large component of Phaeophyta cover (> 50% during certain times) that was not present in the deep habitats, where Chlorophyta was dominant. This was further exemplified by the five sites on the deep ridge complex in north Palm Beach that were dominated by Chlorophyta and Rhodophyta and had very little Phaeophyta if any. Reefs further south in the Broward region were dominated by Chlorophyta, had less Phaeophyta, and had the highest percentage of Cyanophyta. Thus the macroalgae community, which dominates southeastern Florida’s coral reef habitats, varied both latitudinally and across the shelf, providing support for regional separation.

Comparisons of the coral communities also support regional separation. Monitoring data of reefs in similar depths approximately 75 km south (Broward County) found 2.8 times more stony coral species [36]. Gilliam et al. (2010) reported 25 species of coral present in 750 m^2^ of survey area, compared to nine found in Martin in 1737 m^2^ of survey area. Similarly the Southeast Florida Coral Reef Evaluation and Monitoring Project (SECREMP), a regional coral reef monitoring program in place since 2003, found 9 species present in Martin compared to 25 species further south [37]. They also reported Martin had the lowest number of species per station (5.8). Coral density was much lower in Martin. In Broward, coral density of 25 monitoring sites was 2.6 m^−^^2^; 8.1 times greater than our density estimates in Martin (0.32 m^−^^2^) [36]. Finally, *Diadema* were more abundant in the Martin County sites than the sites in the other three counties (24 of the total 46 urchins found) [37]. For comparison, the Florida Keys Coral Reef Evaluation and Monitoring Project found a total of 38 coral species with mean coral species richness of 13.6±0.44 (SEM) per site in the Florida Keys and 19.2±0.84 in the Dry Tortugas [38].

This is not to diminish the importance of reef communities in Martin, but rather to place them in context with the rest of the reef tract. In total, a species list of occurrences has logged twenty-two species of hard corals in Martin since the early 1980’s (although it is unknown if all of these still occur locally) (Jeff Beal, pers. comm.). They also host numerous reef fish species at high densities in certain areas including large aggregations of Goliath grouper (*Epinephelus itajara*) (Walker, pers. obs.).

### 4.2 Nearshore ridge complex cross-shelf patterns

**Figure 6:**
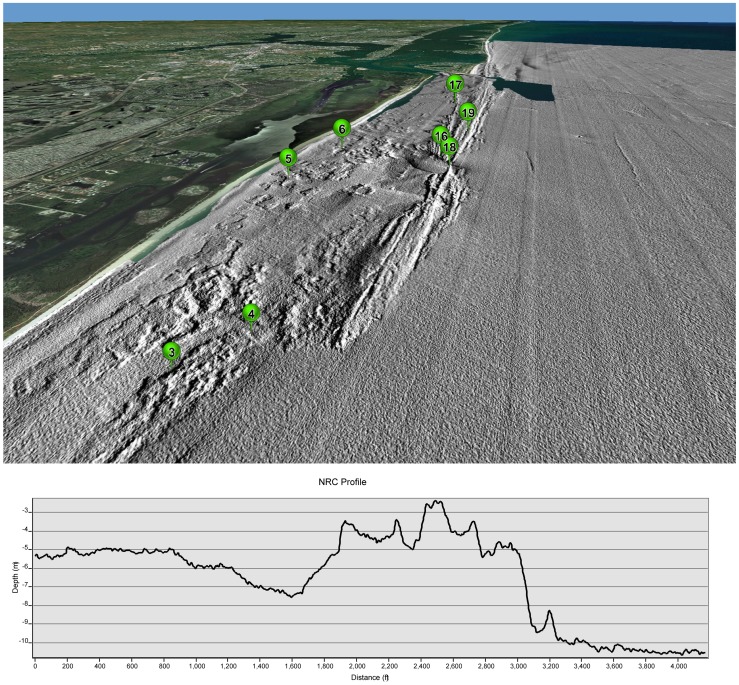
Three-dimensional image of the Nearshore Ridge Complex ((NRC) Ridge-Shallow and Colonized Pavement-Shallow habitats) south of St. Lucie inlet with the quantitative groundtruthing site locations. The depth profile shows a cross-shelf surface contour of the flatter colonized pavement on the left (west), the ridge right of center, and the sand on the right (east). The ridge in this area exhibits a 7 m drop in elevation over an 800 ft distance. Data sources: Grey hillshaded Lidar were created in this study. Imagery includes NASA Blue Marble: Next Generation 500 m resolution imagery at small scales and i-cubed 15 m eSAT imagery at medium-to-large scales for the world. The map also includes i-cubed Nationwide Prime 1 m or better resolution imagery for the contiguous United States. I-cubed Nationwide Prime is a seamless, color mosaic of various commercial and government imagery sources, including Aerials Express 0.3 to 0.6 m resolution imagery for metropolitan areas and the best available United States Department of Agriculture (USDA) National Agriculture Imagery Program (NAIP) imagery and enhanced versions of USGS Digital Ortho Quarter Quad (DOQQ) imagery for other areas.

Inspection of the benthic cover MDS plot (Figure 2) exhibited subtler distinctions between sites that might explain the high within-habitat variability on the shallow colonized pavement and ridge habitats. The Nearshore Ridge Complex (NRC), a combination of Ridge-Shallow and Colonized Pavement-Shallow habitats, appeared to have cross-shelf community patterns. Site 19, which was separated from all other sites in the MDS, was located on the eastern side of the shallow ridge and had a distinct community comprised mostly of macroalgae, turf algae, and Palythoa (Figure 6). Sites 16 and 18, which were very similar to each other in the MDS, were associated with the shallowest top portion of the ridge, the crest. All of the other shallow sites (3, 4, 5, 6, and 17) were located on the western side of the shallow ridge crest and grouped in a central axis in the MDS. A depth profile of the NRC shows drastic changes in the seafloor depth over short distances. Going from east to west (as wave energy does), the seafloor rises 7 m in a distance of 800 ft (near site 19) to ∼2 m depth at the crest (Sites 16 and 18). The seafloor then drops down over 4 m on the western side of the ridge (site 17) before rising and flattening out over the shallow colonized pavement (near site 5 and 6). This type of profile is indicative of many shallow reef systems where differences in communities are driven by light, depth, and energy exposure to form fore-reef, reef crest, back-reef, and lagoon communities. It is likely that although the structure is not comprised of coral, the distinct profile is providing different conditions across the shelf that are shaping the benthic communities. This could account for larger within-habitat variations because the shallow ridge was not divided into separate habitats to account for the differences across the fore-ridge, crest, and back-ridge.

### 4.3 Accuracy assessment

The overall accuracy for major habitat was similar to other regional mapping efforts. Overall map accuracy in Martin was less than Broward (89.6%) [1], Palm Beach (89.2%) [16], and Miami-Dade (93.0%) [17], however it was higher than all of them after adjusting for map marginal proportions. The other mapping efforts did not account for this, but it is an important aspect in Martin County given the disparity between hard and soft bottom areas (95.2% vs. 4.13%). This is much different than Palm Beach (63.9% soft, 35.02% hard), Broward (46.8% soft, 54.2% hard), and Miami-Dade (50.47% soft, 29.65% hard) and likely had a profound effect on the outcome. The map marginal proportion correction was a necessary adjustment in this case and likely better reflects the true map accuracy.

### 4.4 Coral reef range expansion considerations

The benthic habitats in the northern latitudes of the Florida Reef tract off Martin County are distinctly different in both habitat morphology and biological communities than the reefs further south. The shelf is much wider, yet the amount of exposed hardbottom habitat is much less. And, as reported in regional monitoring studies, the number of coral species is reduced 77% from 38 in the Florida Keys to 9. This pattern is similar to other high-latitude reef systems located on eastern continental margins [3], [5], [39], [40], [41].

Although the causes limiting coral reef growth are complex, temperature is often used as a surrogate because of its high correlations with many of the causative factors [3], [42]. Thus climate change has recently been implicated in poleward shifts of tropical coral species, presumably due to increases in temperature from global warming [5], [6]. These shifts have been suggested [7] but not documented for the modern Florida Reef Tract. This study provides a baseline for future comparisons to help determine the effects of global warming in this high-latitude community.

Historic information might give clues as to how present coral reefs may respond to global warming [7]. Historic Holocene FRT growth is evident in SE FL lidar bathymetry [33]. The new Martin County lidar data showed no visual evidence of historic reef growth. Historic reef growth as evidenced by lidar geomorphology ends approximately 31 km south [12], [33], [43]. This historic reef thrived during the Holocene between approximately 8–10,000 years ago [33], [44], [45] on the same deep ridge that extends into Martin County today [12], [33]. For that period, coring data and climate models suggest that yearly mean sea surface temperatures around Florida were warmer (∼2°C) [46] and the climate was much drier (∼0.5 mm/day less precipitation) [47] than the present. Therefore, although historical temperatures were much warmer and coral reefs thrived nearby, they did not extend further northward.

An explanation for the abrupt end to historic coral reef growth might be evident along the coast today.

**Figure 7:**
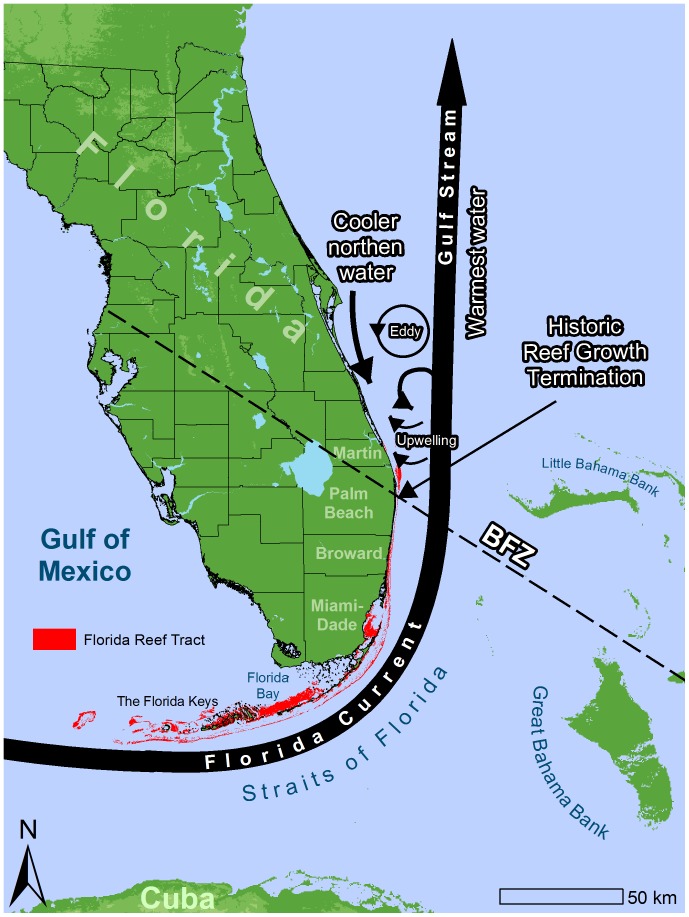
Illustration depicting the hydrodynamics along the southeast Florida coast. A combination of the Florida Current ushering the warmest water offshore, frequent cold water upwelling, and relatively cooler coastal waters off north of Palm Beach County may inhibit a future coral reef poleward shift. Data sources: Land is USGS/EROS Global 30 Arc-second elevation data. Florida Reef Tract is a combination of 2001 FWC-FWRI, NOAA, and Dade County map and the maps created by Walker et al. (2008) and Walker (2012).

Martin County is situated just north of a distinct area along the southeast Florida coast called the Bahamas Fault Zone [48] (Figure 7). This location not only marks the end of historical outer reef growth, but it is also where the shelf widens northward and the Florida Current diverges from the coast [49]. This divergence carries the warmest waters into the Gulf Stream and allows colder northern water to bathe the coast. Here Gulf Stream boundary eddies form and propagate northward along the coast [50], [51] and frequent upwelling occurs [52], [53], [54]. During upwelling events, temperatures can fluctuate by 10°C for days to several weeks [54], [55] and have been implicated as a cause for latitudinal differences in benthic communities [12]. SECREMP reef temperature data show that more than ten such events occurred between February 2007 and May 2009 in the Ridge–Shallow habitat [37].

Globally, the poleward distribution of coral reefs coincides with the 18°C isotherm [42]. Temperatures near 16°C can cause stress in most tropical coral species and lower temperatures can be fatal depending on the duration of exposure [56], [57]. At least five cases of large scale coral mortality have been documented along the Florida Reef Tract since 1960 when temperatures fell below these thresholds [56], [58], [59], [60], [61], [62]. The main corals unaffected by these cold spells were *Siderastraea* and *Oculina*, which are known to be cold tolerant [56], [62]. During our study water temperatures on the deep ridge sites were 15°C for at least several days, which may explain why the Martin hard bottom habitats are dominated by small *Siderastraea* and *Oculina* colonies, neither of which is considered a major constructional component of tropical Caribbean coral reefs.

It is likely that intense, frequent, long-duration upwelling events are inhibiting tropical coral reef communities from establishing in the Martin region. Although it is unknown how climate change will affect coastal currents and upwelling, historic reef growth in Martin during warmer times is not visually evident. If the Gulf Stream continues to carry the warmest water offshore and these upwellings continue, conditions in Martin and further north will not be conducive for coral reef development and may inhibit poleward expansion of tropical coral reefs.
